# Ascidiacea (Chordata: Tunicata) of Greece: an updated checklist

**DOI:** 10.3897/BDJ.4.e9273

**Published:** 2016-11-01

**Authors:** Chryssanthi Antoniadou, Vasilis Gerovasileiou, Nicolas Bailly

**Affiliations:** ‡ Department of Zoology, School of Biology, Aristotle University of Thessaloniki, Thessaloniki, Greece; § Institute of Marine Biology, Biotechnology and Aquaculture, Hellenic Centre for Marine Research, Heraklion, Greece

**Keywords:** Sea-squirts, Aplousobranchia, Phlebobranchia, Stolidobranchia, Aegean Sea, Levantine Sea, Ionian Sea, eastern Mediterranean

## Abstract

**Background:**

The checklist of the ascidian fauna (Tunicata: Ascidiacea) of Greece was compiled within the framework of the Greek Taxon Information System (GTIS), an application of the LifeWatchGreece Research Infrastructure (ESFRI) aiming to produce a complete checklist of species recorded from Greece. This checklist was constructed by updating an existing one with the inclusion of recently published records. All the reported species from Greek waters were taxonomically revised and cross-checked with the Ascidiacea World Database.

**New information:**

The updated checklist of the class Ascidiacea of Greece comprises 75 species, classified in 33 genera, 12 families, and 3 orders. In total, 8 species have been added to the previous species list (4 Aplousobranchia, 2 Phlebobranchia, and 2 Stolidobranchia). Aplousobranchia was the most speciose order, followed by Stolidobranchia. Most species belonged to the families Didemnidae, Polyclinidae, Pyuridae, Ascidiidae, and Styelidae; these 4 families comprise 76% of the Greek ascidian species richness. The present effort revealed the limited taxonomic research effort devoted to the ascidian fauna of Greece, which is attributed to the lack of experts and low sampling effort. Therefore, major knowledge gaps on the ascidian diversity of Greece occur and further research in this field is needed.

## Introduction

The class Ascidiacea (phylum Chordata, subphylum Tunicata) is globally represented by over 2,800 marine species (Shenkar and Swalla 2011, Shenkar et al. 2016b). Ascidians have been identified as a distinct zoological group since the ancient times; Aristotle was the first who described these peculiar animals, "*Téthya*", as the most extraordinary ones having a completely hidden body inside a leathery shell attached on rocks and with two openings some distance apart (Voultsiadou and Vafidis 2007). Their taxonomic classification proved to be challenging for zoologists. As members of the subphylum Tunicata, their close affinity to vertebrates has been recently confirmed by phylogenomic studies (Delsuc et al. 2006). Furthermore, the original classification of Lahille (1886), which classified the ascidian species into the orders Aplousobranchia, Phlebobranchia, and Stolidobranchia according to the structure of the branchial sac, is supported by molecular phylogeny and is currently accepted by most taxonomists (Shenkar and Swalla 2011). However, there are still different views on the placement of several families into orders, and the phylogenetic relationships within the ascidians remain fuzzy (Turon and López-Legentil 2004, Moreno et al. 2008, Pérez-Portela et al. 2009, Tsagkogeorga et al. 2009, Shenkar et al. 2016a).

The first list of Mediterranean ascidians (Pérès 1958, Pérès 1967) reported 132 species; since then, the relevant scientific research increased leading to 229 ascidian species at present (Coll et al. 2010, Shenkar and Swalla 2011). Most records are from the western Mediterranean (165 species), where much more effort has been devoted as opposed to the eastern basin, from which only 86 species have been reported (Koukouras et al. 1995). Among Mediterranean ascidians, 103 exclusive species are included and the entire basin has been recognized as an area of endemism, at least for this specific taxonomic group (Naranjo et al. 1998, Moreno et al. 2014).

In the Greek seas, however, only scattered records of ascidian species were available, either in relevant faunistic accounts (e.g. Hartmeyer 1904, Monniot and Monniot 1974, Koukouras and Siamidou-Efremidou 1978) or in general ecological publications (e.g. Pérès and Picard 1958, Kiseleva 1963, Vamvakas 1971). Koukouras et al. (1995) published the first checklist of the Aegean Ascidiacea, covering also the eastern Mediterranean basin and the Black Sea. So far, this work was the only comprehensive systematic account on the Greek ascidians, combining primary data with an exhaustive literature review. After this publication, a number of studies have been conducted covering various aspects of ascidian biology, such as population dynamics (Panagiotou et al. 2007, Panagiotou et al. 2008, Vafidis et al. 2008), reproduction (Panagiotou et al. 2008, Vafidis et al. 2008), fisheries (Antoniadou and Vafidis 2008), and ecology in general (Morri et al. 1999, Antoniadou et al. 2006, Antoniadou et al. 2013, Sini et al. 2014) or focusing on ascidian associations with other invertebrates (Voultsiadou et al. 2007, Voultsiadou et al. 2010). This research effort has led to some new records of ascidian species in the Aegean Sea (i.e. Morri et al. 1999, Sini et al. 2014), in parallel with additional biodiversity records (Thessalou-Legaki et al. 2012) and the presence of non-indigenous species (Kondilatos et al. 2010, Katsanevakis et al. 2014).

Therefore, the aim of the present work is to compile an updated checklist of Ascidiacea of the Greek seas. For this purpose, the earlier list compiled by Koukouras et al. (1995) has been extended, updated, and annotated according to the recent literature and taxonomic status.

## Materials and methods

The Checklist of Ascidiacea of Greece (Suppl. material 1) was compiled within the context of the Greek Taxon Information System (GTIS). GTIS is an application of the LifeWatchGreece Research Infrastructure (ESFRI) aiming to produce a complete inventory of the Greek biota, by joining relevant efforts. As a first step, the publication of Preliminary Checklists for each taxonomic group has been suggested (Bailly et al. 2016). The present checklist of ascidians has been based on the key-publication of Koukouras et al. (1995), who had compiled the ascidian species list of the Aegean Sea for the first time. In the course of the current study, all recent primary literature was also thoroughly searched and the relevant data were incorporated into the updated species list. New species additions are annotated in the checklist and cited along with the first literature reference reporting their presence in the Greek seas. Non-indigenous species (NIS) are also marked. A cross-checking of all species names and their higher classification was carried through the Ascidiacea World Database, AWD (Shenkar et al. 2016b); the classification followed in the present checklist is the one proposed by the AWD, which contains an updated list of all ascidian species, recursively revised by ascidian taxonomy experts (Shenkar and Swalla 2011).

## Checklists

### Checklist of Ascidiacea known to occur in Greek waters

#### Ascidiacea

#### Aplousobranchia

#### Clavelinidae

#### Clavelina dellavallei

(Zirpolo, 1825)

#### Clavelina lepadiformis

(Müller, 1776)

#### Didemnidae

#### Didemnum amourouxi

Lafargue, 1976

#### Didemnum coriaceum

(Drasche, 1883)

#### Didemnum drachi

Lafargue, 1975

#### Didemnum fulgens

(Milne Edwards, 1841)

#### Didemnum granulosum

(Drasche, 1883)

#### Didemnum maculosum

(Milne Edwards, 1841)

#### Didemnum peyrefittense

Brément, 1913

#### Diplosoma listerianum

(Milne Edwards, 1841)

##### Notes

Recorded by Morri et al. (1999)

#### Diplosoma spongiforme

(Giard, 1872)

#### Lissoclinum perforatum

(Giard, 1872)

##### Notes

Recorded by Thessalou-Legaki et al. (2012)

#### Polysyncraton bilobatum

Lafargue, 1968

#### Polysyncraton lacazei

(Giard, 1872)

#### Trididemnum cereum

(Giard, 1872)

#### Trididemnum inarmatum

(Drasche, 1883)

#### Polycitoridae

#### Cystodytes dellechiajei

(Della Valle, 1877)

#### Eudistoma costai

(Della Valle, 1877)

#### Polycitor crystallinus

(Renier, 1804)

#### Polyclinidae

#### Aplidium aegeaensis

(Hartmeyer, 1904)

#### Aplidium albicans

(Milne Edwards, 1841)

#### Aplidium asperum

Drasche, 1883

#### Aplidium conicum

(Olivi, 1792)

#### Aplidium elegans

(Giard, 1872)

##### Notes

Recorded by Sini et al. (2014)

#### Aplidium nordmanni

(Milne Edwards, 1841)

#### Aplidium pallidum

(Verrill, 1871)

#### Aplidium pseudolobatum

(Pérès, 1956)

#### Aplidium turbinatum

(Savigny, 1816)

#### Aplidium undulatum

Monniot & Gaill, 1978

#### Polyclinella azemai

Harant, 1930

#### Polyclinum aurantium

Milne Edwards, 1841

##### Notes

Recorded by Morri et al. (1999)​

#### Pseudodistoma cyrnusense

Pérès, 1952

#### Phlebobranchia

#### Ascidiidae

#### Ascidia colleta

Monniot C. & Monniot F., 1970

#### Ascidia mentula

Müller, 1776

#### Ascidia muricata

Heller, 1874

#### Ascidia salvatoris

(Traustedt, 1885)

#### Ascidia virginea

Müller, 1776

#### Ascidiella aspersa

(Müller, 1776)

#### Ascidiella scabra

(Müller, 1776)

#### Phallusia fumigata

(Grube, 1864)

#### Phallusia mammillata

(Cuvier, 1815)

#### Phallusia nigra

Savigny, 1816

##### Notes

Recorded by Kondilatos et al. (2010); NIS

#### Cionidae

#### Ciona intestinalis

(Linnaeus, 1767)

#### Ciona roulei

Lahille, 1887

##### Notes

Recorded by Thessalou-Legaki et al. (2012)

#### Corellidae

#### Corella parallelogramma

(Müller, 1776)

#### Rhodosoma turcicum

(Savigny, 1816)

#### Diazonidae

#### Diazona violacea

Savigny, 1816

#### Rhopalaea neapolitana

Philippi, 1843

#### Perophoridae

#### Ecteinascidia turbinata

Herdman, 1880

##### Notes

Recorded by Monniot (1983)

#### Perophora listeri

Wiegman, 1835

#### Stolidobranchia

#### Molgulidae

#### Eugyra arenosa

(Alder & Hancock, 1848)

#### Molgula appendiculata

Heller, 1877

#### Molgula manhattensis

(De Kay, 1843)

#### Molgula occulta

Kupffer, 1875

#### Pyuridae

#### Halocynthia papillosa

(Linnaeus, 1767)

#### Herdmania momus

(Savigny, 1816)

##### Notes

Recorded by Katsanevakis et al. (2014); NIS

#### Microcosmus claudicans

(Savigny, 1816)

#### Microcosmus nudistigma

Monniot C., 1962

#### Microcosmus polymorphus

Heller, 1877

#### Microcosmus sabatieri

Roule, 1885

#### Microcosmus savignyi

Monniot, 1962

#### Microcosmus vulgaris

Heller, 1877

#### Pyura dura

(Heller, 1877)

#### Pyura microcosmus

(Savigny, 1816)

#### Pyura squamulosa

(Alder, 1863)

#### Pyura tessellata

(Forbes, 1848)

#### Styelidae

#### Botryllus schlosseri

(Pallas, 1766)

#### Botrylloides leachii

(Savigny, 1816)

#### Distomus variolosus

Gaertner, 1774

#### Polycarpa caudata

Monniot C. & Monniot F., 1974

#### Polycarpa fibrosa

(Stimpson, 1852)

#### Polycarpa gracilis

Heller, 1877

#### Polycarpa pomaria

(Savigny, 1816)

#### Styela canopus

(Savigny, 1816)

#### Styela plicata

(Lesueur, 1823)

## Discussion

A total of 75 species classified, according to AWD, into 33 genera, 12 families and 3 orders, have been reported from the Greek seas. In addition, *Ascidia conchilega*, Müller, 1776 which is included in AWD and WoRMS databases is a doubtful record for the Greek seas because its original reference for the area is missing and thus it was not included in the list by Koukouras et al. (1995). Aplousobranchia and Stolidobranchia are the most speciose orders with 32 and 25 species, respectively. Aplousobranchia reach 36 species, if the families Diazonidae and Cionidae are included therein instead of Phlebobranchia, as suggested by various ascidian taxonomists based on morphological, developmental, and molecular data (Kott 1990, Turon and López-Legentil 2004, Shenkar and Swalla 2011, Shenkar et al. 2016a) but not currently adopted by AWD. The families Didemnidae (14 species), Polyclinidae (12 species), Pyuridae (12 species), Ascidiidae (10), and Styelidae (9 species) comprised the highest number of species, covering altogether 76% of the Greek ascidian species richness. The first two families have been identified as the most speciose ones across most Mediterranean longitudinal bands (Moreno et al. 2014).

Most ascidian species included in the present checklist were already known as elements of the Greek fauna (Koukouras et al. 1995), while 8 new additions were made in the course of this compilation. These additions include: 4 species of Aplousobranchia, namely *Aplidium elegans*, *Diplosoma listerianum*, *Lissoclinum perforatum*, and *Polyclinum aurantium*; 2 species of Phlebobranchia, namely *Ciona roulei* and *Ecteinascidia turbinata*; and 2 species of Stolidobranchia, namely *Herdmania momus* and *Phallusia nigra*. Although the latter species may need additional confirmation because of the identification uncertainty between the 3 darkly pigmented *Phallusia* species when based on exclusively external features (Vandepas et al. 2015), we consider its presence as valid since it has been reported from the south sector of the Aegean Sea by multiple authors (Kondilatos et al. 2010, Çinar 2014).

The Greek ascidian fauna is mainly composed by species of Atlanto-Mediterranean origin (44.4%) or by endemic species (40.3%), as previously suggested (Koukouras et al. 1995). Among the newly added species, *C. roulei* is a Mediterranean endemic, whereas *Phallusia nigra* and *Herdmania momus* are considered non-indigenous species (NIS) of circum(sub)tropical and Indo-pacific origin, respectively (Gerovasileiou et al. 2016); both species have been probably introduced through shipping (Galil et al. 2016). Another three species, i.e. *Molgula occidentalis* Traustedt, 1883, *Pycnoclavella nana* (Lahille, 1890), and *Microcosmus exasperatus* Heller, 1878, have been recently reported from the Turkish coasts of the Aegean (Çinar 2014); this may imply their presence in the nearby Greek coasts, since they are part of the same ecoregion, the Aegean Archipelago (Spalding et al. 2007) and also part of the Levantine cluster, i.e. areas highly impacted by Erythraean species migration (Galil et al. 2016). Finally, we should mention the presence of *Symplegma brakenhielmi* (Michaelsen, 1904) in the Turkish levantine coasts (Çinar et al. 2006). Since several non-indigenous ascidians, such as *P. nigra* and *H. momus*, have invaded the Greek waters few years after their first record in Turkish waters, it is reasonable to expect them to be reported from south Greece as well, within next few years.

The present updated checklist of Ascidiacea of the Greek seas summarizes the status of the current knowledge. However, a major gap in our knowledge on ascidian diversity of the Greek seas is obvious when the number of species is compared to those known from the western Mediterranean. This can be attributed to two main reasons: (i) the absence of ascidian expertise in Greece and, (ii) the fact that the entire literature on Greek ascidians refers almost exclusively to the Aegean Sea. The necessity for further research on ascidian diversity becomes therefore obvious and expands to the Ionian and Levantine coasts of Greece, from which the ascidian fauna has practically not at all been studied so far.

## Supplementary Material

Supplementary material 1 Checklist of the ascidian fauna (Tunicata: Ascidiacea) of Greece Data type: Taxonomic checklist
Brief description: Taxonomic checklist of Ascidiacea known to occur in Greek waters.
File: oo_97915.xls Chryssanthi Antoniadou, Vasilis Gerovasileiou, Nicolas Bailly

XML Treatment for Ascidiacea

XML Treatment for Aplousobranchia

XML Treatment for Clavelinidae

XML Treatment for Clavelina dellavallei

XML Treatment for Clavelina lepadiformis

XML Treatment for Didemnidae

XML Treatment for Didemnum amourouxi

XML Treatment for Didemnum coriaceum

XML Treatment for Didemnum drachi

XML Treatment for Didemnum fulgens

XML Treatment for Didemnum granulosum

XML Treatment for Didemnum maculosum

XML Treatment for Didemnum peyrefittense

XML Treatment for Diplosoma listerianum

XML Treatment for Diplosoma spongiforme

XML Treatment for Lissoclinum perforatum

XML Treatment for Polysyncraton bilobatum

XML Treatment for Polysyncraton lacazei

XML Treatment for Trididemnum cereum

XML Treatment for Trididemnum inarmatum

XML Treatment for Polycitoridae

XML Treatment for Cystodytes dellechiajei

XML Treatment for Eudistoma costai

XML Treatment for Polycitor crystallinus

XML Treatment for Polyclinidae

XML Treatment for Aplidium aegeaensis

XML Treatment for Aplidium albicans

XML Treatment for Aplidium asperum

XML Treatment for Aplidium conicum

XML Treatment for Aplidium elegans

XML Treatment for Aplidium nordmanni

XML Treatment for Aplidium pallidum

XML Treatment for Aplidium pseudolobatum

XML Treatment for Aplidium turbinatum

XML Treatment for Aplidium undulatum

XML Treatment for Polyclinella azemai

XML Treatment for Polyclinum aurantium

XML Treatment for Pseudodistoma cyrnusense

XML Treatment for Phlebobranchia

XML Treatment for Ascidiidae

XML Treatment for Ascidia colleta

XML Treatment for Ascidia mentula

XML Treatment for Ascidia muricata

XML Treatment for Ascidia salvatoris

XML Treatment for Ascidia virginea

XML Treatment for Ascidiella aspersa

XML Treatment for Ascidiella scabra

XML Treatment for Phallusia fumigata

XML Treatment for Phallusia mammillata

XML Treatment for Phallusia nigra

XML Treatment for Cionidae

XML Treatment for Ciona intestinalis

XML Treatment for Ciona roulei

XML Treatment for Corellidae

XML Treatment for Corella parallelogramma

XML Treatment for Rhodosoma turcicum

XML Treatment for Diazonidae

XML Treatment for Diazona violacea

XML Treatment for Rhopalaea neapolitana

XML Treatment for Perophoridae

XML Treatment for Ecteinascidia turbinata

XML Treatment for Perophora listeri

XML Treatment for Stolidobranchia

XML Treatment for Molgulidae

XML Treatment for Eugyra arenosa

XML Treatment for Molgula appendiculata

XML Treatment for Molgula manhattensis

XML Treatment for Molgula occulta

XML Treatment for Pyuridae

XML Treatment for Halocynthia papillosa

XML Treatment for Herdmania momus

XML Treatment for Microcosmus claudicans

XML Treatment for Microcosmus nudistigma

XML Treatment for Microcosmus polymorphus

XML Treatment for Microcosmus sabatieri

XML Treatment for Microcosmus savignyi

XML Treatment for Microcosmus vulgaris

XML Treatment for Pyura dura

XML Treatment for Pyura microcosmus

XML Treatment for Pyura squamulosa

XML Treatment for Pyura tessellata

XML Treatment for Styelidae

XML Treatment for Botryllus schlosseri

XML Treatment for Botrylloides leachii

XML Treatment for Distomus variolosus

XML Treatment for Polycarpa caudata

XML Treatment for Polycarpa fibrosa

XML Treatment for Polycarpa gracilis

XML Treatment for Polycarpa pomaria

XML Treatment for Styela canopus

XML Treatment for Styela plicata
